# Assessing the Effect of the U.S. Vaccination Program on the Coronavirus Positivity Rate With a Multivariate Framework

**DOI:** 10.1029/2022GH000771

**Published:** 2023-06-06

**Authors:** A. Sanchez‐Vargas, J. Mendez‐Astudillo, Y. López‐Vidal, D. López‐Carr, F. Estrada

**Affiliations:** ^1^ Institute of Economic Research National Autonomous University of Mexico Mexico City Mexico; ^2^ Programa de Inmunología Molecular Microbiana Departamento de Microbiología y Parasitología Faculty of Medicine National Autonomous University of Mexico Mexico City Mexico; ^3^ Department of Geography University of California, Santa Barbara Santa Barbara CA USA; ^4^ Instituto de Ciencias de la Atmósfera y Cambio Climático National Autonomous University of Mexico Mexico City Mexico; ^5^ Institute for Environmental Studies Vrije Universiteit Amsterdam Amsterdam The Netherlands; ^6^ Programa de Investigación en Cambio Climático National Autonomous University of Mexico Mexico City Mexico

**Keywords:** factors feedback and simultaneity, U.S. vaccination program, complex relationships, error correction models, machine learning, time series and panel data

## Abstract

The factors influencing the incidence of COVID‐19, including the impact of the vaccination programs, have been studied in the literature. Most studies focus on one or two factors, without considering their interactions, which is not enough to assess a vaccination program in a statistically robust manner. We examine the impact of the U.S. vaccination program on the SARS‐CoV‐2 positivity rate while simultaneously considering a large number of factors involved in the spread of the virus and the feedbacks among them. We consider the effects of the following sets of factors: socioeconomic factors, public policy factors, environmental factors, and non‐observable factors. A time series Error Correction Model (ECM) was used to estimate the impact of the vaccination program at the national level on the positivity rate. Additionally, state‐level ECMs with panel data were combined with machine learning techniques to assess the impact of the program and identify relevant factors to build the best‐fitting models. We find that the vaccination program reduced the virus positivity rate. However, the program was partially undermined by a feedback loop in which increased vaccination led to increased mobility. Although some external factors reduced the positivity rate, the emergence of new variants increased the positivity rate. The positivity rate was associated with several forces acting simultaneously in opposite directions such as the number of vaccine doses administered and mobility. The existence of complex interactions, between the factors studied, implies that there is a need to combine different public policies to strengthen the impact of the vaccination program.

## Key Points

- Mobility and vaccination are key factors in reducing COVID‐19 positivity rate, they both have a feedback effect on each other
- Socioeconomic factors, public policy factors, environmental factors, and non‐observable factors and their interactions affect positivity rate
- A multivariate model shows complex relationships between vaccination and positivity rate

## 1 Introduction

In March 2020 the World Health Organization declared the start of the COVID‐19 pandemic due to the novel SARS‐CoV‐2 (Fauci et al., 2020). Since then, the COVID‐19 pandemic has caused disruptions around the world (He et al., 2020). The spread of the disease has been monitored with the positivity rate which considers the daily number of tests and confirmed cases during the time interval (Kafle et al., 2021). The severity of the disease once contracted is positively related to a number of factors, including a compromised immune system, age, and obesity (Shang et al., 2020). In order to contain the spread of the disease, authorities from around the world implemented lockdowns and other mitigation actions that included mobility restrictions, social distancing measures (de Souza Melo et al., 2021), and confinements (López & Rodó, 2020) which led to fewer infections and deaths (Hsiang et al., 2020).

Other factors worldwide that have been associated with COVID‐19 incidence are environmental variables such as mean air temperature (Méndez‐Arriaga, 2020), humidity (Mecenas et al., 2020), and precipitation (Liu et al., 2020). Also, air quality and Particulate Matter concentrations have been associated with the spread of the disease and smoking has been linked to increased disease severity (Guo, 2020). For example, in England, a positive relation between air pollution concentration and deaths associated with COVID‐19 was found (Travaglio et al., 2021). A positive relation between Nitrogen Dioxide (NO_2_) and COVID‐19 fatalities was found with data from Italy, Spain, France, and Germany (Zhu et al., 2020). In China, a positive relation was found between air pollutant concentrations and SARS‐CoV‐2 infections (Zhu et al., 2020).

In the United States of America (USA) there is evidence of a wide range of factors that played an important role in COVID‐19 incidence, including the effect of the U.S. vaccination program (Moghadas et al., 2021). Also, a positive relationship between COVID‐19 incidence and population mobility in US has been previously reported with SARS‐CoV‐2 detection by PCR (Abouk & Heydari, 2021).

In 2021 as waves of the pandemic advanced, vaccines were made available and vaccination programs were implemented worldwide (Ortiz & Neuzil, 2022). In the literature, the effect of vaccination campaigns against the original viral variant in US has been assessed with randomized controlled trials, mathematical simulations, and epidemiological models (Alagoz et al., 2021; Dagan et al., 2021). An inverse relation between the vaccination program and the number of SARS‐CoV‐2 infections has been established (Chodick et al., 2022). Some studies suggested that the efficacy of the vaccination program may be enhanced by combining it with other interventions that limit community transmission, such as social distancing, and mask use (López & Rodó, 2020; Ortiz & Neuzil, 2022). However, few studies considered the potential feedback between the main variables associated with the spread of the virus in assessing the effect of the COVID‐19 vaccine. For instance, the vaccination program may affect population mobility and vice versa. A statistical model without such feedbacks among its variables is unlikely to explain much of the variability in the level of the SARS‐Cov‐2 transmission at a country level (Vilches et al., 2021). In general, such papers found that the vaccination program was effective in reducing the COVID‐19 incidence without considering the role played by the interactions among the big set of factors associated to the positivity rate and the vaccination program, which may reduce the effectiveness of the vaccine.

The objective of this paper is to assess the effect of the U.S. vaccination program on the SARS‐CoV‐2 positivity rate, while considering the main factors of the spread of the virus modeling the existing direct and indirect channels between them and the potential feedbacks between them. Socioeconomic factors (mobility and unemployment claims), public policy factors (number of applied vaccines, mobility restrictions), environmental factors (temperature, humidity, precipitation, air quality), and non‐observable factors (vaccine preference, vaccine hesitancy) were controlled and analyzed using a two‐stage empirical strategy. Previous studies in the literature include fewer factors in their studies. Therefore, we believe the importance of our study is that it can be used to learn the importance of direct, indirect, and feedback effects when designing a response to future pandemics caused by Coronaviruses or air‐borne viruses. First, a cointegrated (Simultaneous Equations [SE]) system, at the national level, was estimated by using time series data (Johansen & Juselius, 2009). Second, the same cointegrated methods, at the state level, were applied by using a large panel database to account for geographical, public policy, air quality and climatic differences across states and to verify the statistical robustness of our national results. Specifically, the Pooled Mean Group (PMG) estimator with a large panel database of 49 states was used, over the 1 January 2021–23 January 2022, period, with weekly data. Panel data analysis techniques allowed the authors to consider unobserved heterogeneity constant over time (i.e., time invariant preferences for the vaccine at the state level) (Blackburne & Frank, 2007). They also allowed to control for a wider set of observed factors associated with the positivity rate at the same time.

## 2 Materials and Methods

Socioeconomic factors, public policy factors, environmental factors, and non‐observable factors affecting COVID‐19 propagation in US were assessed using a multivariate framework and an empirical framework. By controlling for such factors, we aim to properly isolate the effect of the vaccination program on the positivity rate.

### 2.1 Multivariate Framework

A multivariate framework was implemented to study the effect of several factors affecting the positivity rate of COVID‐19. The potential relationships among the main variables associated with the positivity rate and feedback loops among them was described by a system of three SE which represent long‐run relationships among the variables. They contain three endogenous variables (the SARS‐CoV‐2 positivity rate, the population mobility, and the number of vaccine doses administered to people) and a wide set of other relevant factors at the national and state levels, determined from outside the model (weather, air quality, and policy interventions). The definition of the system of equations is described in detail in Supporting Information S1.

The assessment of the total effect of the U.S. vaccination program on positivity rate was done by taking the total derivative of *y*_*it*_ (the positivity rate for state *i* in week *t*) with respect to *x*_1*it*_ (the number of doses administered to people) as defined in Equation 1:

(1) $$\frac{d{yi}_{t-1}}{dx_{1it-1}}=\left(\alpha +\beta \zeta \right)<0,$$

Equation 1 implies that the vaccination program would reduce the positivity rate when α>βζ. This may happen if the effect of the vaccination program on the positivity rate (*α*) is greater than the effect of a higher population mobility on the positivity rate (*β*) multiplied by the effect of the vaccination program on population mobility (*ζ*). Equation 1 also suggests that there are forces that move in opposite directions, which policy makers need to consider when designing successful public health policies to face the COVID‐19 pandemic. Thus, the total effect of the vaccination program is an empirical issue that can be resolved by estimating the signs of the parameters in the system with real data.

### 2.2 Empirical Approach

The parameters of the three long‐run equations, at the national level, were estimated with a time series data cointegrated SE system (Johansen & Juselius, 2009; Menebo, 2020; Vilches et al., 2021). Then, the statistical robustness of the cointegrated SE time series model was verified by estimating the three proposed equations, at the state level to capture he effect of heterogeneity, using panel Error Correction Methods (ECMs) with a large panel data set (Blackburne & Frank, 2007).

**Figure 1:**
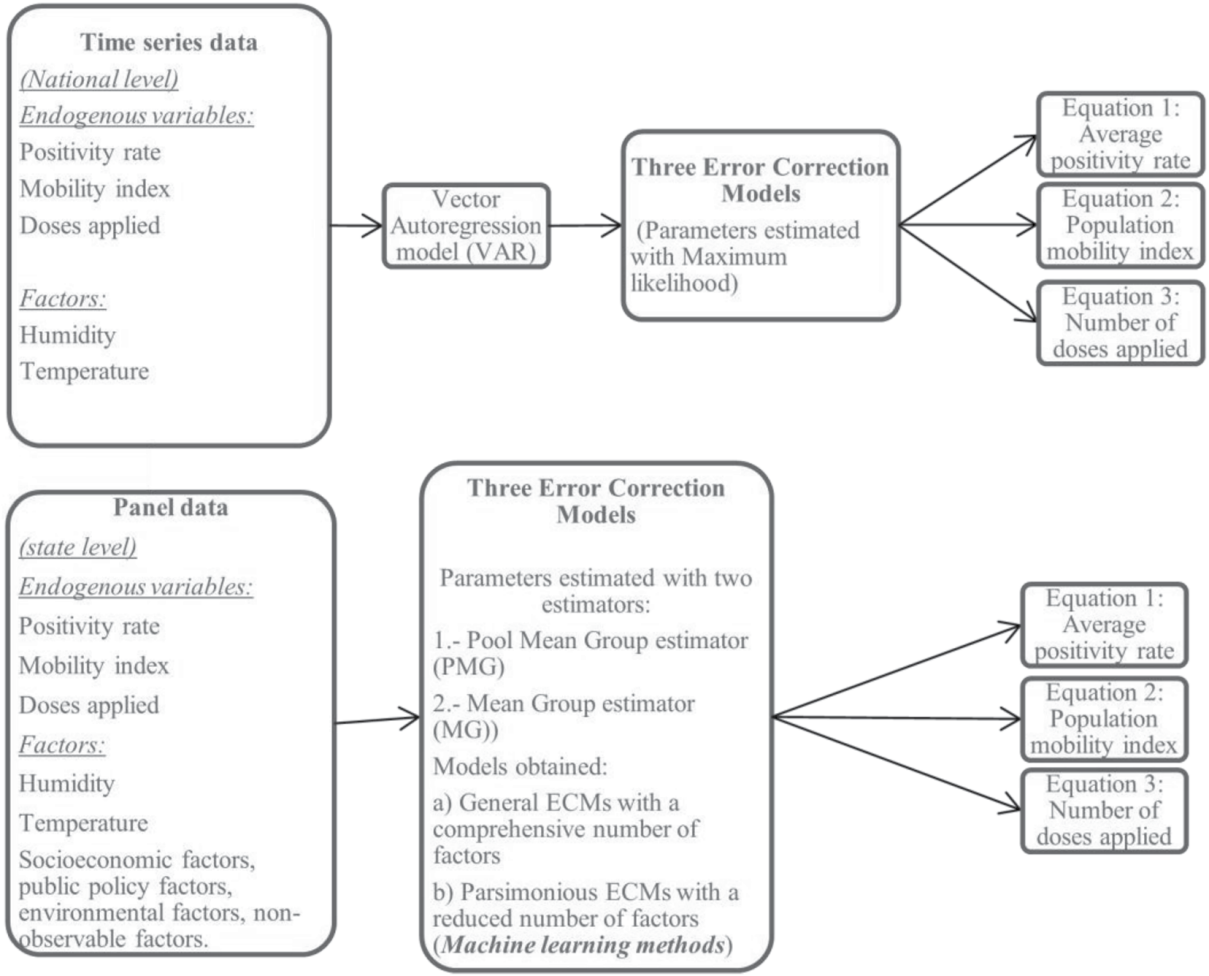
Flowchart of the methodology of this study.

The methodology developed for this study is shown in Figure 1.

**Figure 2:**
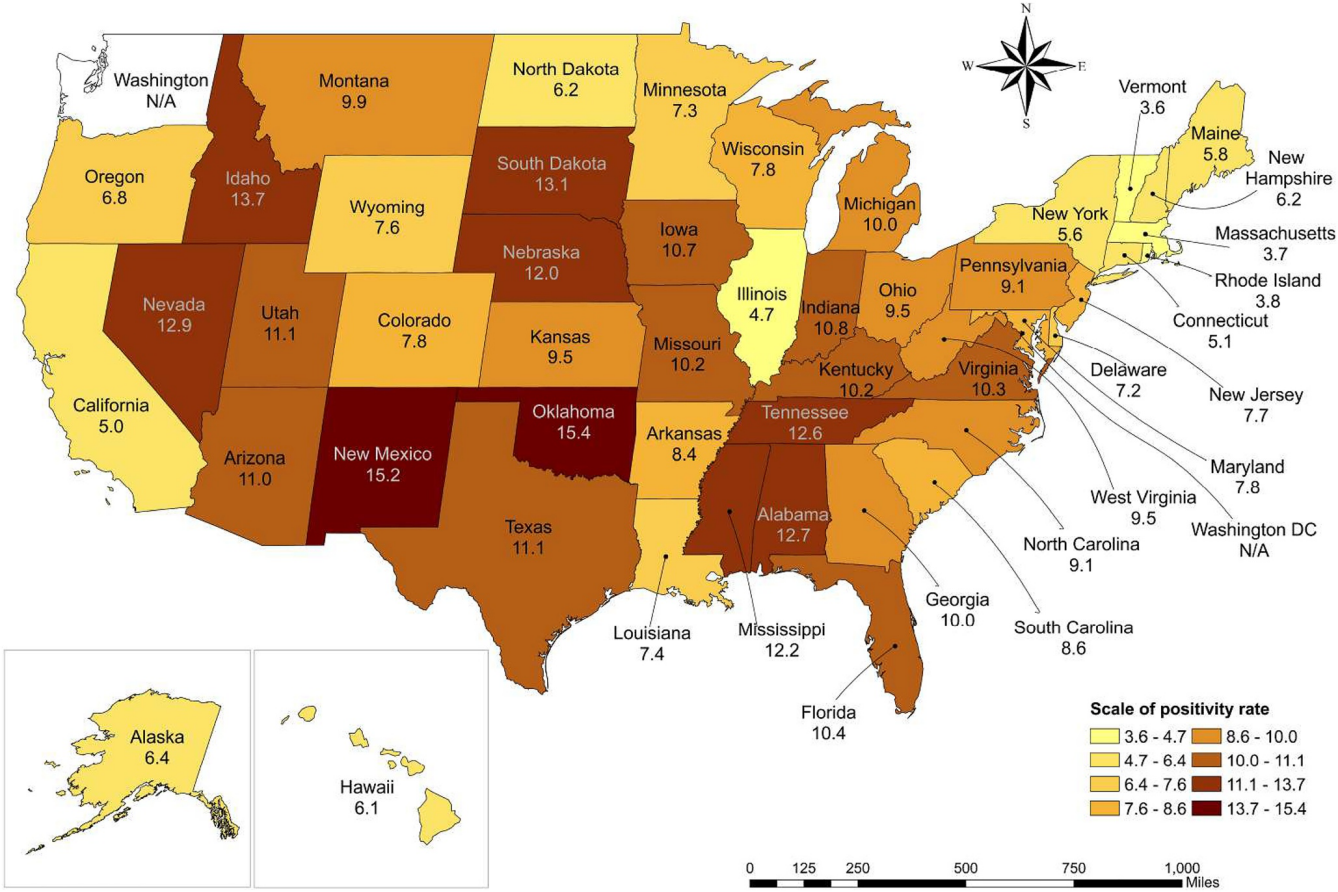
Average positivity rate from 1 January 2021 to 23 January 2022 in USA.

A panel Error Correction (EC) model was estimated by using two different estimators that allowed the authors to consider different forms of heterogeneity in its parameters across states (Blackburne & Frank, 2007). For example, average positivity, number of doses applied, and average mobility index in the different states as shown in Figure 2 and Figures S1 and S2 in Supporting Information S1, respectively.

The Pool Mean Group estimator (PMG) restricted the long‐run parameters of the long‐run effects to be equal across states but allowed the short‐term effects of the model to be state‐specific. Also, the Mean Group estimator (MG) was used because it allowed the model to have completely heterogenous long‐run and short‐run parameters. The Hausman test was used to choose the appropriate estimator (PMG or MG) for our data. The empirical approach is explained in detail in Supporting Information S1.

Due to the possibility of including a wider number of factors in a panel Error Correction Model (ECM), two versions of the estimated models are proposed: (a) a comprehensive panel ECMs with all the available factors and (b) a more parsimonious panel ECMs with a reduced number of relevant factors, which are selected by using machine learning techniques such as the Lasso, Ridge, and Elastic Net regression models.

## 3 Data

For the cointegrated SE model, weekly time series from the first week of January 2021 to the third week of January 2022 were used. Specifically, the model included US positivity rate (CDCb, 2022), the population mobility index (CDC, 2022), and the number of doses administered to people (CDCa, 2022). These data were published by the Centers for Disease Control and Prevention (CDC). The time series model also contained the average temperature and average relative humidity (NOAA, 2022) available at the National Oceanic and Atmospheric Administration's web site (NOAA).

For the panel data ECMs at the state‐level, a large panel database with a total of 3,136 observations was used, resulting from 54 weeks of data for 49 states (with the exception of Washington DC), during the same time span. This database included the level, first, second, and third lags of the first differences of the variables. Furthermore, the level and lags of the first difference of the percentage of fully vaccinated people over time was included as a factor. Moreover, some dichotomous variables were built using information from the CDC such as: (a) variables indicating the timeline of the emergence of COVID‐19 new variants (CDCc, 2022) such as Delta, Epsilon, Eta, Mu, and Omicron, (b) variables representing the vaccine rollout timeline for specific age groups (young people older than 18 years, adolescent older than 12 years and children older than 5 years), and (c) other dichotomous variables indicating the existence of public policy restrictions such as mask use, gathering bans, stay at home restrictions, and restaurant and bar closures. Furthermore, other variables were included such as the air quality index (AQI) and particulate matter (PM_2.5_) concentrations at the state level (EPA, 2022), published by the U.S. Environmental Protection Agency. Additionally, the models contained the unemployment insurance weekly claims (U.S. Department of Labor Employment and Training Administration, 2022), which are published by the U.S Department of Labor. A detailed description of the variables used in this study can be found in Table S1 in Supporting Information S1. Unemployment is an important variable because it was a direct consequence of the restrictions, and it has potentially contributed to excess deaths in US (Matthay et al., 2021). All data was processed with Stata (Python, 2022; StataCorp LLC, 2022).

## 4 Results

### 4.1 Empirical Evidence From a Cointegrated SE System With Time Series Data at the National Level

Unit root tests performed on the data showed that the 7‐day average percent positivity rate, the number of doses of vaccines administered, a population mobility index, the average temperature and average relative humidity are first‐difference stationary. A Vector Autoregression (VAR) model with these variables and five lags was estimated. A Cointegration Rank test performed to the VAR model indicated the existence of three cointegrating vectors among our five variables, at the 95% confidence level. This is fully consistent with the hypothesis on the existence of three long‐run equations. Then, we reparameterized the VAR model as a system of three cointegrated SE: (a) the SARS‐CoV‐2 positivity rate, (b) the population mobility, and (c) the number of vaccine doses administered to people (Johansen & Juselius, 2009). Next, the cointegrated SE system was estimated using the Full Information Maximum Likelihood method.

The results in the first column of Table 1 suggest that the positivity rate went down when the number of doses administered went up (−6.06). In contrast, the positivity rate was higher when there was an increase in population mobility (0.84). Moreover, according to the results, population mobility decreased with a higher positivity rate (−1.32). The simultaneous relationship between population mobility and the number of vaccines is also shown in Table 1. That is, population mobility increased when the number of vaccines went up (6.94), whereas the number of doses seemed to increase when there was a higher population mobility (0.08). The estimated coefficients reflected only the existence of positive or negative long‐run associations among our variables. Therefore, a variance decomposition analysis was carried out to obtain results in term of percentage changes. The variance decomposition analysis for the 7‐day average positivity rate is shown in Table S2 in Supporting Information S1.

**Table 1:** The Normalized Cointegrating Vectors as Long‐Run Equations for the 7‐Day Average Positivity Rate, Population Mobility Index, and the Number of Doses Administered to People in US From January 2021 to January 2022

	Seven‐day average positivity rate	Population mobility index	Log of the number of doses
Long‐run coefficients			
Seven‐day average positivity rate	1.000	−1.32	0.05
		(0.33)	(0.01)
		[3.97]	[3.89]
Log of the number of doses	−6.06	6.94	1.000
	(1.17)	(2.96)	
	[5.17]	[2.34]	
Population mobility index	0.84	1.000	0.08
	(0.24)		(0.02)
	[3.51]		[3.61]
Average temperature	−0.79	0.39	−0.08
	(0.14)	(0.08)	(0.01)
	[5.60]	[4.83]	[6.37]
Average relative humidity	0.000	0.69	−0.08
		(0.46)	(0.02)
		[1.50]	[4.73]

*Note.* Log, logarithm. The standard errors are reported in parenthesis and the *p*‐value is reported in square brackets, *p* < 0.05 considered statistically significant.

**Figure 3:**
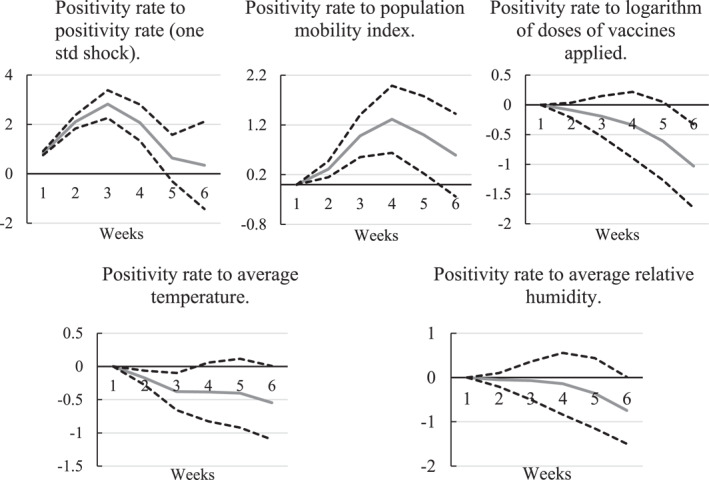
Impulse responses to Cholesky One S.D. of the positivity rate to standard deviation shock in USA from January 2021 to January 2022. Abbreviation: Log, logarithm.

Figure 3 shows the impulse responses of the positivity rate to one standard deviation (std) shocks (an increase of one standard deviation) in the current positivity rate, population mobility, the number of doses given to people, average temperature, and humidity.

Panel 1 of Figure 3 suggests that a shock of 1 week in the positivity rate generated, a higher level of COVID‐19 infections. Panel 2 of Figure 3 suggests that a shock to population mobility caused the positivity rate to increase sharply, starting in week 2. That is, population mobility had an increasing effect on the number of SARS‐CoV‐2 infections. A shock in the number of doses administered to people led to a continuous decrease in the positivity rate, starting in the second week. This result implies that the vaccination program needs some time to have a positive impact among the population. Time is needed to have more than 50% of the population vaccinated against SARS‐CoV‐2 and to build immunity against the coronavirus infection. The pandemic has shown that the vaccination campaigns helped to build hybrid immunity where, despite being vaccinated, patients still acquired the SARS‐CoV‐2 infection with weaker effects.

In summary, the cointegrated SE system suggested the potential existence of the expected relationships among the variables chosen in this study, as implied in the basic framework. However, the estimated coefficients can be considered only indicative results since they were based on a time series model with only a few variables and a limited number of observations. These results do not account for geographical factors and other time‐invariant unobserved differences at the state level such as the preferences for the vaccine. Thus, in the next section, panel data EC models were estimated to control for such differences and ensure the statistical validity and robustness of the time series results at the national level.

### 4.2 Empirical Evidence From Cointegrated Panel Data EC Models at the State Level in US

Table 2 shows the long‐run parameters estimated by using panel ECMs with only 45 relevant variables, which were selected from a larger set of 57 factors with machine learning techniques (see Elastic net, Lasso, and Ridge regression results in Table S4 in Supporting Information S1). As a sensitivity test, the same three models were estimated with the larger set of 57 variables and similar results were found (Table S5 in Supporting Information S1).

**Table 2:** Estimations of Nonstationary Heterogeneous Panels for US From January 2021 to January 2022

	Panel A (Equation S1 in Supporting Information S1: 7‐day average positivity rate)			Panel B (Equation S2 in Supporting Information S1: Population mobility index)			Panel C (Equation 3 in Supporting Information S1: Number of doses applied)		
	PMG	MG	Joint H‐stat	PMG	MG	Joint H‐stat	PMG	MG	Joint H‐stat
Machine learning models									
Long‐run coefficients									
Log of number of doses administered to people	−3.59*** (0.44)	−5.24* (2.95)	8.45 [0.999]	0.28*** (0.08)	−0.12 (0.22)	10.67 [0.9965]			6.10 [0.998]
Population mobility index	1.94*** (0.19)	0.55 (3.32)					0.07** (0.03)	−0.64 (0.49)	
Average temperature				−0.008** (0.003)	−0.02** (0.01)		−0.04*** (0.008)	0.11 (0.17)	
Average relative humidity	−1.10*** (0.07)	−0.56 (0.69)		0.09*** (0.016)	0.08*** (1.052)		−0.12*** (0.02)	0.27 (0.27)	
Constant	−2.31*** (0.32)	11.60 (6.16)		18.26*** (1.44)	24.86*** (2.12)				
Error correction coefficient	−0.22*** (0.01)	−0.27*** (0.02)		−0.39*** (0.03)	−0.59*** (0.06)		−0.11*** (0.01)	−0.17*** (0.01)	
No. Obs	2,695			2,695			2,744		
No. States	49			49			49		

*Note.* Log, logarithm; PMG, Pooled Mean Group estimator; MG, Mean Group estimator; and Joint H‐stat, Durbin‐Wu‐Hausman Test. The standard errors are reported in parenthesis and probability value of the *z* score, with *P* < 0.05 considered significant. The superscripts ⁎⁎⁎, ⁎⁎, and ⁎ indicate significant at 1%, 5%, and 10% levels, respectively. Machine Learning models Long‐run coefficient.

A large panel data set of 49 states for 54 weeks (from January 2021 to January 2022) was used to estimate the long‐run parameters and also to control for a wider set of observable factors by including them in the short‐run structure of the models. Moreover, we were able to control for all the unobservable factors by using state‐specific fixed effects. We included in our model the first, second, and third lags of the first differences of the factors associated with the positivity rate, population mobility, and the number of doses administered.

Furthermore, the three Panel EC models were estimated with the PMG, and the MG estimators and valid estimates were chosen with the Hausman test. The first, second, and third lags of the first differences of the factors associated with the positivity rate (population mobility and the number of doses administered) were included in the model.

The results of our models are shown in Table 2. According to the Hausman test in columns 3, 6, and 9 of Table 2 (Joint‐H stats), the null hypothesis of homogeneous long‐run parameters at the state level cannot be rejected for the three equations. Therefore, the consistent and efficient PMG estimates (columns 1, 4, and 7) are the preferred ones over the MG estimates (columns 2, 5, and 8). The PMG estimator restricts the long‐run slope parameters of the model to be equal across states, as if there were only one positivity equation at the national level but allows the short‐term parameters to vary at the state level. This result makes the long‐run results of our panel ECM model comparable with the results of the time series cointegrated SE system.

In column 1 of panel A, the estimated panel ECM for the positivity rate is reported. The positivity rate is negatively associated with the number of doses administered to people (−3.59), Thereby confirming that the U.S. vaccination program successfully reduced the positivity rate at the national level in the long run. On the other hand, population mobility increased the positivity rate (1.94) in the long‐run equation. It was also found that a higher relative humidity (−1.10) contributed to reduce the positivity rate. In contrast to the times series model, the average temperature was not found to be statistically significant in the long‐run structure of the positivity rate ECM model.

In column 1 of panel B, it is shown that the number of vaccines (0.280) increased the population mobility in the long run. In contrast with the time series cointegrated SE system, there was not statistically significant long‐run relationship between population mobility and the positivity rate. However, this relationship did exist, with the same sign, in the very short term (−0.07) (Table S3 in Supporting Information S1).

In the first column of panel C, we report a statistically significant long‐term relationship between the number of doses administered to people and the population mobility. Thereby confirming that there existed feedback between those two factors. A statistically significant relationship between the number of doses and the positivity rate in the long run was not found, although the short‐run equation for the number of doses suggested a positive short‐term relationship between them. Both, long‐ and short‐run results are presented in detail in Table S6 in Supporting Information S1.

According to the results presented in Table S6 in Supporting Information S1, the positivity rate is negatively associated to increase of current and lagged precipitation (−3.57 and −8.0). Also, the positivity rate is negatively associated to the increase of the lagged vaccination coverage (−0.61) and to the approval of the vaccine for specific age groups, such as adolescents older than 12 years old (−0.82). Furthermore, it was negatively associated to the rise of unemployment insurance (−0.45). Conversely, the positivity rate was positively associated with the emergence of new variants of the virus such as Delta (0.60), Epsilon (0.71), Eta (0.53), and Omicron (2.52), and the increase of particulate matter concentration (0.02).

The population mobility index was negatively associated to the AQI and to the positivity rate (−0.07) in the short run. The panel ECMs also allowed us to control for unobserved effects that do not vary over time using state‐level fixed effects estimates. Specifically, we were able to control for unobservable preferences for the vaccine ‐at the state level‐ in the equation for the number of doses given to people.

## 5 Discussion

Table 1 shows that population mobility increased when the number of vaccines went up and the number of doses applied seemed to increase when there was a higher population mobility, similar results were found in Spain (Briz‐Redón & Serrano‐Aroca, 2020). The estimated coefficients reflected only the existence of positive or negative long‐run associations among our variables. Therefore, a variance decomposition analysis was carried out to obtain results in term of percentage changes. The variance decomposition analysis for the 7‐day average positivity rate is shown in Table S2 in Supporting Information S1.

According to the variance decomposition analysis, most of the variations in positivity rate are explained by its own shocks after 2 weeks. However, other factors started to play an important role after 3 weeks. After 7 weeks, 62.77% of the variations in the positivity rate were explained by internal shocks and 10.89% by the shocks to the number of doses administered to people. The combination of population mobility, the number of doses administered to people, and the climatic factors explained 37.21% of the variation in the forecast error for the positivity rate after 7 weeks, which was in line with the previous empirical findings (Dave et al., 2021). These figures suggested that the U.S. vaccination program was successful in reducing the positivity rate, however, a combination of other factors influenced it. Thus, a proper analysis of the impact of the vaccination campaign on the COVID‐19 incidence must consider the role of different forces playing roles in opposite directions and in direct and indirect ways.

**Figure 4:**
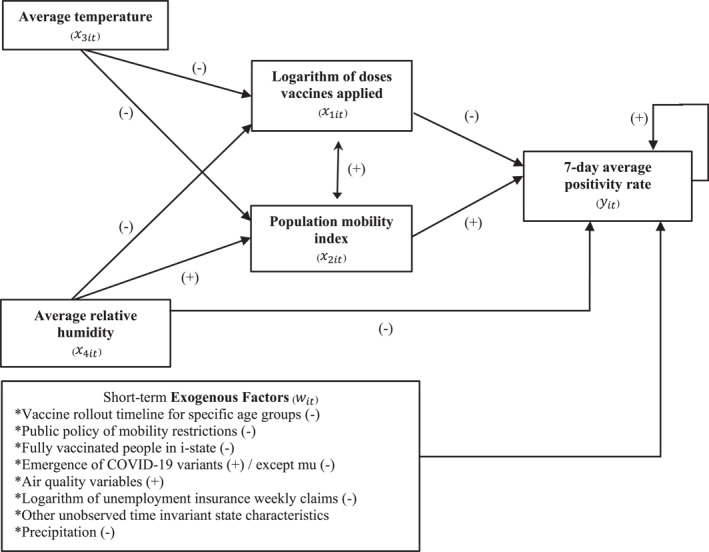
Interactions between the variables in the system of equations.

Figure 4 summarizes the main empirical findings of this study. It shows the statistically significant long‐run associations among the variables in the proposed model, including the feedback between population mobility and the number of SARS‐CoV‐2 vaccine doses. Furthermore, in the bottom square, the short‐term exogenous factors are included.

Figure 4 suggests that the number of vaccine doses administered to people can affect the positivity rate through the following two channels: (a) through its direct effect on the positivity SARS‐CoV‐2 rate and (b) through its effect on population mobility which, in turn, affects the rate of confirmed SARS‐CoV‐2 infections. Therefore, the SARS‐CoV‐2 vaccination program directly reduces the positivity rate, but the vaccination program may also raise the positivity rate by increasing population mobility due to people going out once they are vaccinated. These two effects may offset each other and reduce the total effect of the vaccination program.

Vaccination had a positive effect on mobility, therefore, mobility increased when the vaccination rate increased. The increase in vaccination rates was a reason for relaxing mobility restrictions. Thus, mobility increased, and positivity increased in the short‐term. However, one of the main effects of full vaccination is the reduction of hospitalization or deaths caused by the SARS‐CoV‐2 virus and allowing to re‐start economic and social activities.

The results displayed in Table S3 in Supporting Information S1 show that in the short‐run, the emergence of new COVID‐19 variants had a significant positive effect on positivity rate. This means that when a new variant emerged, the positivity rate increased and made the effect of the vaccination less significant until the vaccines or the immune systems of the patients were adapted to the new variant. Therefore, vaccine boosters were included in vaccination programs. Also, the results show that public policies do not significantly affect the vaccination rate, then, vaccination and public policies can be seen as complementary measures to control the spread of the pandemic.

All effects presented in Figure 3 can be direct or indirect. Average humidity has a direct effect on the positivity rate as well as an indirect effect on the positivity rate through its effects on the number of vaccine doses applied and population mobility. Also, average humidity has a direct positive effect on mobility (mobility seems to increase when humidity increases), and mobility has a positive effect on 7‐day average positivity rate (the more people move, the more infections). However, the direct effect between humidity and positivity is negative which might seem a contradiction. The model was built using partial derivatives; thus, the total effect is the sum of the partial effects. In this case, the relation between humidity and positivity rate will depend on the weights of each of the partial effects which depends on local characteristics of the population and habits. Therefore, ambiguous relations between humidity and positivity rate have been reported in the literature (Mecenas et al., 2020).

High temperatures (Sasikumar et al., 2020) and air pollution concentrations (Chattopadhyay & Shaw, 2021) have been related to COVID‐19 spread and deaths, respectively. Therefore, we included these factors in the model and temperature was found to have a negative relation to number of doses applied and mobility index. A possible interpretation is that when temperatures rose, people would rather do other activities than to get vaccinated, also it may be that during hot days people prefer to stay indoors. Air quality was not a significant factor in this model because it is related to deaths rather than contagions (Chattopadhyay & Shaw, 2021).

Mobility restrictions help to reduce contagions; however, small droplets of the virus can remain airborne for long periods of time, and they can travel considerable distances. Therefore, when vaccinated or unvaccinated people inhale these droplets, they can acquire the infection. It is estimated that droplets in the air can contain 10^6^–10^9^ virus/ml for the SARS‐CoV‐2 virus (Oswin et al., 2022) Relative humidity between 20% and 100% can impact the transmission of the virus. One percent reduction of positivity rate is found between 40% and 80% of relative humidity (Oswin et al., 2022). Also, the seasonal periodicity of respiratory infections in humans needs to be considered.

The results of the short‐term model presented in Table S3 in Supporting Information S1, shows that there is a direct negative relation between public policies and mobility. This means that public policies were implemented to reducing mobility and mobility reduction is related to a positivity rate reduction. Therefore, according to the results of this paper, public policies had an indirect effect on positivity rate through mobility. Mobility is highly related to positivity rate (Boto‐García, 2023).

Given the significant effect of mobility on positivity rate, the model included other variables affecting mobility such as weekly unemployment insurance claims. In this case, the results show that unemployment is not a significant factor affecting mobility which means that other factors such as stay‐home orders or work from home have a higher influence on mobility. A marginal effect of unemployment on mobility was found with a 2‐week delay.

The results of this study suggest that policies implemented to contain the pandemic should consider the interactions between the factors here described and should focus on mobility control. Thus, policy makers should pay attention to trends in population mobility in order to avoid a higher number of infections, at least until total herd immunity is achieved. In order to reduce transmission when the transmission dynamics of SARS‐CoV‐2 is high, social distancing, mask wearing mandates, and complete vaccination schemes need to be implemented. These are lessons that can be learn in case a new pandemic from a coronavirus or from an air‐borne virus arises in the future.

The limitation of this study is that only data from 2021 was used once the original variant passed. Furthermore, in 2021 some mobility restrictions were already lifted so our study only includes the effect of those remaining restrictions. Also, some effects here described are valid only for USA given its social and economic characteristics. Future work could include data from different parts of the world or include information about vaccine brand or maker.

## 6 Conclusion

A multivariate framework was built to assess the effect of the U.S. vaccination program on the positivity rate of the SARS‐CoV‐2 virus, which was formalized as a set of three SE. Its empirical validity was tested by estimating the parameters of such equations in the long‐run structure of a cointegrated SE system. The impulse response functions and variance decomposition analysis for the positivity rate were also estimated in this study.

The use of a cointegrated SE system allowed the authors to consider a wider set of factors associated with the positivity rate and the feedbacks among them. This modeling approach is useful to obtain an unbiased estimate of the effect of the vaccination program and a better understanding of the role played by different factors on the positivity rate. However, the model here presented has the limitation that the results are valid only through the period January 2020–January 2021. As the pandemic advanced, the virus mutated and the attitudes toward restrictions changed so, these changes would have to be considered in future models.

The results suggest that a higher number of doses administered to people reduces the positivity rate. However, the effect of the U.S. vaccination program seems to be partially offset by the combined effect of higher population mobility and dry weather. Also, statistically significant feedback was found between the vaccination program and population mobility, which may partly reduce the effect of the vaccination program. These findings suggest that population mobility, geographical and meteorological factors, and their interactions, must be considered to obtain a proper estimate of the effect of vaccination program in any statistical model. Such factors and their interactions must also be considered when designing public policies to cope with potential new waves of the SARS‐Cov‐2 virus.

Policy makers should pay particular attention to the trends in population mobility in order to avoid a higher number of infections. Specifically, policy makers and government officials might usefully adjust mobility restrictions according to trends in population mobility. While it is difficult for the western world to apply such stringent mandates as exemplified by China's zero COVID tolerance policy, especially in the regards to controlling human mobility, policy makers should be aware that with increased mobility, infection rises and be prepared to implement tried and true measures. These include social distancing and mask wearing when the transmission dynamics of SARS‐CoV‐2 is high. Finally, complete vaccination schemes should be promoted to reduce transmission.

## Conflict of Interest

The authors declare no conflicts of interest relevant to this study.

## Supporting information

Supporting Information S1

## Data Availability

Air Quality data are available in https://www.epa.gov/outdoor-air-quality-data/air-quality-index-daily-values-report. The population mobility index are available in https://covid.cdc.gov/covid-data-tracker/#mobility. The number of doses of vaccines applied are available in https://covid.cdc.gov/covid-data-tracker/#trends_weeklycases_totalvaccinesadministered_00. The total number of positive cases in US are available in https://covid.cdc.gov/covid-data-tracker/#trends_weeklycases_totalcasesper100k_00. The timeline of the emergence of variants is available in https://www.cdc.gov/coronavirus/2019-ncov/variants/variant-classifications.html?CDC_AA_refVal=https:%2F%2Fwww.cdc.gov%2Fcoronavirus%2F2019-ncov%2Fvariants%2Fvariant-info.html. All of them are made available by the Center of Disease Control and Prevention (CDC). Temperature, humidity, and precipitation data are available in the National Oceanic and Atmospheric Administration's site: https://www.ncei.noaa.gov/cdo-web/datatools/lcd. The number of weekly unemployment insurance claims are available in https://oui.doleta.gov/unemploy/claims.asp. All data was processed with STATA17 and Python 3.
