# Supplementary material for: Assessing the Effect of the U.S. Vaccination Program on the Coronavirus Positivity Rate With a Multivariate Framework
Source: Geohealth. 2023 Jun 6;7(6):e2022GH000771. doi: 10.1029/2022GH000771 (PMC10243209; doi:10.1029/2022GH000771)
Supplement: Supplementary file 1 — Supporting Information S1 [file GH2-7-e2022GH000771-s001.docx]

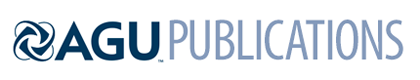


*GeoHealth*

Supporting Information for

**Assessing the effect of the U.S. vaccination program on the Coronavirus positivity rate with a multivariate framework**

A. Sanchez-Vargas^1^, J. Mendez-Astudillo^1^, Y. López-Vidal^2^ , D. López-Carr^3^ and F. Estrada^4,5,6^

^1^Institute of Economic Research, National Autonomous University of Mexico. Mexico City

^2^Programa de Inmunología Molecular Microbiana. Departamento de Microbiología y Parasitología, Faculty of Medicine, National Autonomous University of Mexico. Mexico City.

^3^Department of Geography, University of California, Santa Barbara, California, USA.

^4^Instituto de Ciencias de la Atmósfera y Cambio Climático, National Autonomous University of Mexico. Mexico City.

^5^Institute for Environmental Studies, Vrije Universiteit Amsterdam, Amsterdam, 1081HV, The Netherlands.

^6^Programa de Investigación en Cambio Climático, National Autonomous University of Mexico. Mexico City.

Institutional affiliations

**Contents of this file**

Text S1 to S3

Tables S1 to S6

Figures S1 and S2

**Introduction**

As supporting information, we present three texts and four tables. The texts describe in detail the derivation of the three simultaneous equations used for the study presented in the paper. Also, a text detailing the derivation of the empirical model is included as supporting information. Moreover, the interpretation of short-term effects found with panel data is clearly described. The tables here presented show in detail the variables used in this study and detailed results of the analysis of data used in the study.

Text S1.

Multivariate Framework

In the proposed model, y_it_ is the positivity rate for state *i* in week *t.* The number of doses administered to people are defined as (x_1it_), the population mobility index (x_2it_), the average temperature (x_3it_), and the average relative humidity (x_4it_). The positivity rate may also be associated with a vector of other exogenous observed factors ($w_{it}$) such as: the vaccine rollout timeline for specific age groups (time of the vaccine approval for different age groups), other health public policy restrictions (for example, mobility restrictions, mask use, social distancing measures, bans on gatherings, restaurant and bar closures), the percentage of fully vaccinated people at the state level over time, the timeline of the emergence of new COVID-19 variants, the unemployment insurance weekly claims, the amount of precipitation, the air quality, and particulate matter (PM_2.5_) concentration as follows:

$y_{it-1}={\alpha x}_{1it-1} +{\beta x}_{2it-1}+{\gamma x}_{3it-1}+ {\delta x}_{4it-1}+ w_{it-1}^{´}\varphi+\mu_{i}+ \epsilon_{1it}$ (1),

where$\alpha$, $\beta, \gamma$,$\delta$, and $\varphi$ are unknown parameters and $\mu_{i}$ is a vector of state-specific effects that captures the effect of unobserved time invariant differences among states (i.e., preferences for the vaccine). Moreover, $y_{1it,} x_{1it,}$and $x_{2it}$are endogenous variables since there could be some feedback among them. $x_{3it,}x_{4it,}$ and $w_{it}$are considered predetermined variables, since they do not depend on the other variables included in our model. A long run cointegrated SE system with lagged endogenous variables in equation (1) is used to estimate the unknown parameters as a dynamic system.

We propose to estimate an equation of population mobility (x_2it_), which may be affected by the number of doses administered to people (x_1it_), the average temperature (x_3it_), relative humidity (x_4it_), and other exogenous factors such as air quality, precipitation, ${(w}_{it}^{´})$ and state fixed effects ($\mu_{i}$). With this equation, we consider the potential feedback between the U.S. vaccination program and the population mobility:

$x_{2it-1}=\zeta x_{1it-1}+{\eta x}_{3it-1}+{\theta x}_{4it-1}+w_{it}^{´}\varsigma+\mu_{i}+ \epsilon_{2it}$ (2),

where $\zeta$, $\eta,$ $\theta$, and $\varsigma$ are the parameters to be estimated. Population mobility may be affected by the SARS-CoV-2 positivity rate; however.

The vaccination program (x_1it_) cannot be considered a pure exogenous health intervention, because health authorities may decide to increase the number of vaccines after looking at the level of population mobility ${(x}_{2it})$or the local weather variables${(x}_{3it}, x_{4it}$). Additionally, the number of vaccines administered can also be affected by other factors related to vaccine administration such as the government´s approval of the vaccine for specific age groups ${(w}_{it}^{´})$:

$x_{1it-1}=\kappa x_{2it-1}+{\lambda x}_{3it-1}+{\omega x}_{4it-1}+w_{it}^{´}\xi+\mu_{i}+ \epsilon_{3it}$ (3),

where $\kappa,$ and $\lambda$, $\omega$, and $\xi$ are parameters to be estimated. Again, the state fixed effects ($\mu_{i})$ allowed us to account for state-specific unobserved factors that may not vary over time (i.e., vaccine hesitancy, preferences for the vaccine, government preferences for the vaccine, and other time invariant state-level characteristics).

Text S2.

Empirical framework

As a first step, we apply unit root tests to all our variables in levels to determine if they are non-stationary time series. Then we estimate a Vector Autoregressive model (VAR) for the variables in levels, included in the vector $z_{it}=[y_{it}$, $x_{1it}, x_{2it}, x_{3it}, x_{4it}, w_{it}^{´}]$, which is a dynamic system because the current values of the series depend on their own lags and the lags of the other variables:

$z_{t}={\pi_{1}z}_{t-1}+\pi_{2}z_{t-2}+\ldots+\pi_{q}z_{t-q}+\tau_{t}$ (5),

where the optimal number of lags (*q*) is chosen by using a lag length criterion such as the Hannan Quinn Information Criterion. Next, we use this VAR model to carry out the Cointegration Rank Test to investigate the existence of cointegration among our time series and to determine the number of cointegrating relationships that may be embedded in the system. We then re-parametrize the VAR model as a VECM (Vector Error Correction Model) because the variables are first-difference stationary and share common stochastic trends (Equation 6).

$\Gamma\left( L \right)\Delta z_{it}=\mu+\alpha\beta^{'}z_{it-1}+\tau_{it}$ (6),

The VECM contains the lags of the variables in first differenced form in its short run structure$( \Gamma\left( L \right)\Delta z_{it} )$ and one or more linear combinations of the variables in levels ($\alpha\beta^{'}z_{it-1}$) (cointegrating equations)^1^ .Next, we used the Full Information Maximum Likelihood Method to simultaneously estimate the equations of the VECM as a cointegrated SE system. In estimating this system, we select the number of cointegrating relationships embedded in the long-run structure of the model ($\alpha\beta^{'}z_{it-1}$) based on the cointegration rank test. At the same time, we normalized the cointegrating vectors as equations for some of our endogenous variables (i.e., equations for the positivity rate and other endogenous variables). Finally, we assessed the statistical validity of the model (Equation 6) by using classic diagnostic checks.

Panel data techniques allowed us to check if our results were sensitive to the use of more variables at the state level, more data points, and different estimation methods that account for state-level fixed effects (accounting for geographical, climatic, and other state-level non observable differences across states). As a preliminary step, we specify an Autoregressive Distributed Lagged Regression model (ADLR)

$y_{it}=\sum_{n=1}^{q} \lambda_{in}y_{i,t-n}+\sum_{j=1}^{p} \delta_{in}z_{i,t-n}+\mu_{i}+\tau_{it}$ (7),

where, $y_{i,t-n}$ are lags of the dependent variable and $x_{i,t-n}$ are the contemporaneous and lagged values of other exogenous or predetermined factors (lags of the variables). Moreover, $\mu_{i}$ are state-level fixed effects. To estimate this ARDL model, we had to re-parameterize it as a panel data ECM^2^ as follows:

${\Delta y}_{it}=\phi_{i}\left[ y_{i,t-1}-{\theta^{'}}_{i}z_{i,t} \right]+\sum_{n=1}^{q-1} {\lambda*}_{in}{\Delta y}_{i,t-n}+\sum_{n=1}^{p-1} {\delta*}_{in}z_{i,t-n}+\mu_{i}+\tau_{it}$ (8),

where $\phi_{i}=-(1-\sum_{n=1}^{q} \lambda_{in}), \theta_{i}=- \beta_{i}/\phi_{i} ,\beta_{i}$= $\sum_{n=1}^{p} \delta_{in}$,

${\lambda*}_{in}= -\sum_{n=1}^{q} \lambda_{im}, n=1,2,\ldots, q-1$

and

${\delta*}_{in}=-\sum_{m=n+1}^{q} \delta_{im}, n=1,2,...,p-1$.

If the error correction parameter ($\phi_{i})$ in equation (8) is negative and lower than -2, we assume that there is evidence of the existence of a cointegrating relationship (a long-run equation) between the variables involved in the equation. A cointegrating relationship is embedded in the component: $\phi_{i}[y_{i,t-1}-{\theta'}_{i}z_{i,t}]$ of a panel ECM and it may be assimilated to any of our equations (1), (2), and (3), (i.e., an equation for the positivity rate). The short run of the effects in the model ($\sum_{n=1}^{q-1} {\lambda*}_{in}{\Delta y}_{i,t-n}+\sum_{n=1}^{p-1} {\delta*}_{in}z_{i,t-n}$) are then given by the parameters ${\lambda*}_{in}$and ${\delta*}_{in}$in the equation (8)

Text S3.

Column 1 in table S2 shows that most of the variations in the positivity rate are explained by its own shocks after two weeks (97.38 %). However, other factors started playing an important role after three weeks. At the end of 7 weeks, 62.77 % of the variations in the positivity rate were explained by its own shocks and 10.89 % by the shocks to the number of doses administered to people. The combination of population mobility, the number of doses administered to people, and the climatic factors explained 37.21 % of the variation in the forecast error for the positivity rate after seven weeks, which was in line with the previous empirical findings (Briz-Rendón & Serrano-Aroca, 2020). These figures suggested that the U.S. vaccination program was successful in reducing the positivity rate. However, a combination of other factors influenced it. Thus, a proper analysis of the impact of the vaccination campaign on the COVID-19 incidence must consider the role of different forces playing roles in opposite directions and in direct and indirect ways.

Short-run effects

Column 2 of the panel ECMs, reported in Table S3, show the short-run effects obtained with a reduced number of regressors selected with machine learning techniques. In Table S3 the short-run effects of a large set of exogenous and predetermined factors are shown as lags. For instance, we found that the positivity rate is negatively associated to increase of current and lagged precipitation (-3.57 and -8.0). Also, we found that the positivity rate is negatively associated to the increase of the lagged vaccination coverage (-0.61) and to the approval of the vaccine for specific age groups, such as adolescents older than 12 years old (-0.82). Furthermore, it was negatively associated to the rise of unemployment insurance (-0.45). Conversely, the positivity rate was positively associated with the emergence of new variants of the virus such as Delta (0.60), Epsilon (0.71), Eta (0.53), and Omicron (2.52), and the increase of particulate matter concentration (0.02).

We also found that the population mobility index was negatively associated to the air quality index and to the positivity rate (-0.07) in the short run. The panel ECMs also allowed us to control for unobserved effects that do not vary over time using state-level fixed effects estimates. Specifically, we were able to control for unobservable preferences for the vaccine, at the state level, in the equation for the number of doses given to people.

**Table S1.** Description of the variables in the analysis for the period 2020–2022.

| **Variable** | **Definition** | **Unit of measurement** |
| --- | --- | --- |
| $y_{1it}$ | 7-day average positivity rate | Percent |
| $x_{1it}$ | Logarithm of doses vaccines applied | Logarithm |
| $x_{2it}$ | Population mobility index | Percent |
|  | Environmental factors: |  |
| $x_{3it}$ | Average temperature | °F |
| $x_{4it}$ | Average relative humidity | Percent |
| $w_{1it}$ | Vaccine rollout timeline for specific age groups: | Dichotomous variables |
| $w_{1_{1}\mathrm{it}}$ | Young people (older than 18 years) | Binary variable = 1 if CDC approved covid-vaccines for young people, = 0, otherwise |
| $w_{1_{2}\mathrm{it}}$ | Adolescent (older than 12 years) | Binary variable = 1 if CDC approved covid-vaccines for adolescent, = 0, otherwise |
| $w_{1_{3}\mathrm{it}}$ | Children (older than 5 years) | Binary variable = 1 if CDC approved covid-vaccines for children, =0, otherwise |
| $w_{2it}$ | Public policy of mobility restrictions: | Dichotomous variables |
| $w_{2_{1}\mathrm{it}}$ | Use of mask | Binary variable = 1 if mask mandate was in place, = 0, otherwise |
| $w_{2_{2}\mathrm{it}}$ | Gatherings | Binary variable = 1 if gathering ban was in place, = 0, otherwise |
| $w_{2_{3}\mathrm{it}}$ | Stay at home | Binary variable = 1 if people stay at home mandate was in place, = 0, otherwise |
| $w_{2_{4}\mathrm{it}}$ | Restaurant closure | Binary variable = 1 if restaurant closure in place, = 0, otherwise |
| $w_{2_{5}\mathrm{it}}$ | Bar closure | Binary variable = 1 if bar closure in place, = 0, otherwise |
| $w_{3it}$ | Logarithm of fully vaccinated people in any state | Logarithm |
| $w_{4it}$ | Timeline of the emergence of COVID-19 variants | Dichotomous variables |
| $w_{4_{1}\mathrm{it}}$ | Delta | Binary variable = 1 if delta variant started, =0, otherwise |
| $w_{4it}$ | Epsilon | Binary variable = 1 if epsilon variant started, =0, otherwise |
| $w_{4_{3}\mathrm{it}}$ | Eta | Binary variable = 1 if eta variant started, =0, otherwise |
| $w_{4_{4}\mathrm{it}}$ | Mu | Binary variable = 1 if mu variant started, =0, otherwise |
| $w_{4_{5}\mathrm{it}}$ | Omicron | Binary variable = 1 if omicron variant started, =0, otherwise |
| $w_{5it}$ | Logarithm of unemployment claims | Logarithm of number of claims (weekly) |
| $w_{6it}$ | Air quality index | Index |
| $w_{7it}$ | Particulate matter PM_2.5_ | μg/m^3^ |
| $w_{8it}$ | Average precipitation | mm |
|  | Changes over time |  |
| i | 54-time fixed week effects |  |
|  | Geographical factors: |  |
| t | 49 state fixed effects |  |

**Table S2.** Variance decomposition analysis for the 7-day average positivity rate in the U.S. from January 2021 to January 2022.

| **Week** | **7-day average positivity rate** | **Population mobility index** | **Log of number of doses** | **Average temperature** | **Relative humidity** |
| --- | --- | --- | --- | --- | --- |
| *Relative variance of the 7-day average positivity rate* |  |  |  |  |  |
| **1** | 100.00 | 0.00 | 0.00 | 0.00 | 0.00 |
| **2** | 97.38 | 1.85 | 0.15 | 0.56 | 0.05 |
| **3** | 91.07 | 7.35 | 0.31 | 1.20 | 0.53 |
| **4** | 84.03 | 13.46 | 0.76 | 1.55 | 0.13 |
| **5** | 78.23 | 16.63 | 2.32 | 2.12 | 0.67 |
| **6** | 71.29 | 16.46 | 6.32 | 3.10 | 2.81 |
| **7** | 62.77 | 14.91 | 10.89 | 5.08 | 6.33 |
| **8** | 58.78 | 15.54 | 10.98 | 6.74 | 7.94 |
|  |  |  |  |  |  |

Abbreviation: Log, logarithm.

**Table S3.** Estimations of nonstationary heterogeneous panels (PMG) of positivity rate, mobility index and logarithm of vaccine doses applied for U.S^a^ from January 2021 through January 2022 ^b c^.

|  | **Panel A**  (7-day average positivity rate) | | **Panel B**  (Population mobility index) | | **Panel C** ^d^  (Number of doses applied) | |
| --- | --- | --- | --- | --- | --- | --- |
|  | **General** | **Machine learning** | **General** | **Machine learning** | **General** | **Machine learning** |
| **Long-run coefficients**^e^ |  |  |  |  |  |  |
| Log of number of doses administered to people | - 6.55*** (0.46) | - 3.59*** (0.44) | 0.30*** (0.09) | 0.29*** (0.11) |  |  |
| Population mobility index | 1.26*** (0.14) | 1.94*** (0.19) |  |  | 0.15*** (0.02) | 0.06** (0.03) |
| Average temperature |  |  | - 0.01*** (0.00) | - 0.01*** (0.00) | - 0.01** (0.00) | -0.04*** (0.00) |
| Average relative humidity | - 1.27*** (0.07) | - 1.10*** (0.07) | 0.08*** (0.01) | 0.07*** (0.02) | - 0.16*** (0.01) | -0.12*** (0.01) |
| **Short-run coefficients** |  |  |  |  |  |  |
| Error correction | - 0.27*** (0.01) | - 0.23*** (0.01) | - 0.19*** (0.03) | - 0.23*** (0.02) | - 0.20*** (0.00) | - 0.11*** (0.00) |
| ∆ Log of number of doses administered to people | - 1.68*** (0.20) | - 0.52*** (0.16) | 0.91*** (0.16) | 0.69*** (0.14) |  |  |
| ∆ Log of number of doses administered to people _t-1_ | - 1.78*** (0.22) | - 0.44*** (0.16) | - 0.15** (0.06) | - 0.16*** (0.06) | -0.03 (0.02) | 0.01*** (0.01) |
| ∆ Log of number of doses administered to people _t-2_ | - 0.83*** (0.21) |  | - 0.07 (0.07) | - 0.05 (0.06) | 0.00 (0.01) | 0.03 (0.01) |
| ∆ Log of number of doses administered to people _t-3_ | - 0.79*** (0.19) |  | - 0.06* (0.03) |  |  |  |
| ∆ Population mobility index |  |  |  |  | 0.08*** (0.01) | 0.07*** (0.00) |
| ∆ Population mobility index _t-1_ |  |  | - 0.44*** (0.04) | - 0.37*** (0.03) | - 0.01 (0.01) | -0.00*** (0.00) |
| ∆ Population mobility index _t-2_ |  |  | - 0.24*** (0.03) | - 0.16*** (0.02) | -0.05*** (0.01) |  |
| ∆ Population mobility index _t-3_ |  |  | - 0.14*** (0.03) |  | -0.05*** (0.00) |  |
| ∆ 7-day average positivity rate |  |  | - 0.06*** (0.18) | - 0.07*** (0.01) | 0.01*** (0.00) | 0.01*** (0.00) |
| ∆ 7-day average positivity rate _t-1_ | 0.88*** (0.03) | 0.79*** (0.03) | - 0.00 (0.02) | 0.01 (0.02) | 0.00 (0.00) | -0.00 (0.00) |
| ∆ 7-day average positivity rate _t-2_ | - 0.31*** (0.04) | - 0.29*** (0.04) | - 0.01 (0.02) | 0.00 (0.02) | - 0.02*** (0.00) |  |
| ∆ 7-day average positivity rate _t-3_ | 0.32*** (0.06) | 0.17*** (0.05) | 0.01 (0.01) |  | 0.01*** (0.00) |  |
| ∆ Average temperature | 0.01 (0.01) | 0.02* (0.01) | 0.04*** (0.01) | 0.03*** (0.00) | 0.00*** (0.00) |  |
| ∆ Average temperature _t-1_ | 0.05*** (0.01) | 0.05*** (0.01) | 0.05*** (0.00) | 0.04*** (0.00) | 0.00*** (0.00) | - 0.00*** (0.00) |
| ∆ Average temperature _t-2_ | 0.08*** (0.01) | 0.06*** (0.01) | 0.00* (0.00) | 0.00 (0.00) |  |  |
| ∆ Average temperature _t-3_ | 0.09*** (0.01) | 0.09*** (0.01) | - 0.02*** (0.00) |  | 0.00 * (0.00) |  |
| ∆ Average relative humidity | - 0.22*** (0.02) | - 0.18*** (0.01) | - 0.01** (0.00) | - 0.01*** (0.00) | - 0.02*** (0.00) | - 0.01*** (0.00) |
| ∆ Average relative humidity _t-1_ | - 0.09*** (0.01) | - 0.07*** (0.01) | - 0.00 (0.00) | 0.01** (0.00) | - 0.03*** (0.00) | - 0.01*** (0.00) |
| ∆ Average relative humidity _t-2_ | - 0.08*** (0.01) | - 0.08*** (0.01) | - 0.00 (0.00) | 0.01*** (0.00) | - 0.00** (0.00) |  |
| ∆ Average relative humidity _t-3_ | 0.02 (0.01) |  | - 0.01** (0.00) |  |  |  |
| ∆ Average precipitation | - 6.98*** (1.49) | - 3.57*** (0.92) |  |  | - 0.12 (0.13) | - 0.26*** (10.09) |
| ∆ Average precipitation _t-1_ | -11.68*** (1.36) | - 8.00*** (0.66) |  |  | - 0.03 (0.18) | - 0.57*** (0.13) |
| ∆ Average precipitation _t-2_ | - 2.90** (1.19) |  |  |  | - 2.21*** (0.24) | - 1.75** (0.14) |
| ∆ Average precipitation _t-3_ | - 1.93** (0.91) |  |  |  | - 0.87*** (0.06) | - 0.44** (0.50) |
|  |  |  |  |  |  |  |
| Vaccine rollout timeline for specific age groups^f^: |  |  |  |  |  |  |
| Young people older than 18 years old |  |  |  |  | - 0.34*** (0.01) | - 0.23*** (0.01) |
| Adolescents older than 12 years old | - 0.50** (0.20) | - 0.82*** (0.15) |  |  | 0.31*** (0.02) | - 0.02 (0.01) |
| Children older than 5 years old |  |  |  |  | 0.08*** (0.01) | - 0.02*** (0.01) |
|  |  |  |  |  |  |  |
| Public policy of mobility restrictions: |  |  |  |  |  |  |
| Stay at home | - 0.17 (0.31) | - 0.09 (0.18) | - 0.16 (0.12) | - 0.22*** (0.08) |  |  |
| Use of mask | - 0.80 (0.77) | - 0.15 (0.12) | - 0.31** (0.13) | - 0.27*** (0.09) |  |  |
| Gatherings | 0.11 (0.31) | - 0.14 (0.11) | - 0.14* (0.06) | - 0.13*** (0.04) |  |  |
| Restaurant closed | - 0.22* (0.11) | - 0.04 (0.07) | - 0.01 (0.03) | - 0.05 (0.04) |  |  |
| Bar closed | - 0.01 (0.01) | - 0.01 (0.01) | 0.00 (0.00) | 0.00 (0.00) |  |  |
|  |  |  |  |  |  |  |
| ∆ Log of fully vaccinated people in any state _t-1_ | - 0.80 (0.70) | - 0.61*** (0.13) |  |  |  |  |
| ∆ Log of fully vaccinated people in any state _t-2_ | 0.97** (0.40) |  |  |  |  |  |
| ∆ Log of fully vaccinated people in any state _t-3_ | - 0.06 (0.30) |  |  |  |  |  |
|  |  |  |  |  |  |  |
| Timeline of the emergence of COVID-19 variants: |  |  |  |  |  |  |
| Delta | 1.10*** (0.19) | 0.60*** (0.12) |  |  |  |  |
| Epsilon | 0.27 (0.27) | 0.71*** (0.15) |  |  | 0.04** (0.02) |  |
| Eta | 0.53** (0.21) | 0.53*** (0.16) |  |  | 0.35*** (0.03) |  |
| Kappa | 0.31 (0.23) | 0.35** (0.14) |  |  | -0.51*** (0.02) |  |
| Mu | - 0.75*** (0.11) | - 0.44*** (0.09) |  |  | 0.07*** (0.01) |  |
| Omicron | 2.07*** (0.20) | 2.53*** (0.17) |  |  | -0.28*** (0.01) |  |
| Iota | 0.38** (0.16) | 0.15** (0.06) |  |  |  |  |
|  |  |  |  |  |  |  |
| ∆ Log of unemployment insurance weekly claims | - 0.70*** (0.26) | - 0.45** (0.18) |  |  |  |  |
| ∆ Log of unemployment insurance weekly claims _t-1_ | - 0.52** (0.24) |  |  |  |  |  |
| ∆ Log of unemployment insurance weekly claims _t-2_ | - 0.82*** (0.18) |  |  |  |  |  |
| ∆ Log of unemployment insurance weekly claims _t-3_ | 0.02 (0.18) |  |  |  |  |  |
|  |  |  |  |  |  |  |
| ∆ air quality index |  |  | - 0.00 (0.00) | - 0.00 (0.00) |  |  |
| ∆ air quality index _t-1_ |  |  | 0.00 (0.00) | - 0.00* (0.00) |  |  |
| ∆ air quality index _t-2_ |  |  | 0.01*** (0.00) |  |  |  |
| ∆ air quality index _t-3_ |  |  | 0.00 (0.00) |  |  |  |
|  |  |  |  |  |  |  |
| ∆ PM25 _t-4_ | 0.07*** (0.01) | 0.02** (0.01) |  |  |  |  |
| ∆ PM25 _t-5_ | 0.05*** (0.02) | 0.02 (0.01) |  |  |  |  |
|  |  |  |  |  |  |  |
| Constant | -16.90*** (1.20) | - 2.31*** (0.32) | 8.52*** (1.36) | 10.51*** (0.84) |  |  |
|  |  |  |  |  |  |  |
| No. Obs | 2681 | 2681 | 2695 | 2695 | 2744 | 2744 |
| No. States | 49 | 49 | 49 | 49 | 49 | 49 |

Abbreviation: Log, logarithm, ∆, First difference, _t-1,_ first lag, _t-2,_ second lag, _t-3,_ third lag and _t-4,_ fourth lag.

The standard errors are reported in parenthesis and probability value of the z score, with P < .05 considered significant.
The superscripts ⁎⁎⁎, ⁎⁎ and ⁎ indicate significant at 1%, 5% and 10% levels, respectively.

**Table S4** Lasso, Ridge, and Elastic Net for U.S from January 2021 through January 2022.

|  | **Lasso** | **Ridge** | **Elastic Net** |
| --- | --- | --- | --- |
|  | alpha =  1 | alpha =  0 | alpha =  0.967 |
| (Intercept) | -0.047 | -0.122 | -0.047 |
| ∆ Log of number of doses administered to people | 0.079 | 0.269 | 0.079 |
| ∆ Log of number of doses administered to people _t-1_ | 0 | 0.123 | 0 |
| ∆ Log of number of doses administered to people _t-2_ | 0.043 | 0.175 | 0.043 |
| ∆ Log of number of doses administered to people _t-3_ | -0.009 | -0.011 | -0.009 |
| ∆ 7-day average positivity rate _t-1_ | 0.546 | 0.157 | 0.546 |
| ∆ 7-day average positivity rate _t-2_ | -0.024 | 0.047 | -0.024 |
| ∆ 7-day average positivity rate _t-3_ | -0.042 | -0.021 | -0.042 |
| ∆ Population mobility index | -0.224 | -0.199 | -0.224 |
| ∆ Population mobility index _t-1_ | -0.085 | -0.184 | -0.085 |
| ∆ Population mobility index _t-2_ | 0.083 | -3.57e-3 | 0.083 |
| ∆ Population mobility index _t-3_ | 0.026 | 0.010 | 0.026 |
| ∆ Average temperature | -0.002 | -0.020 | -0.002 |
| ∆ Average temperature _t-1_ | -0.001 | -0.008 | -0.001 |
| ∆ Average temperature _t-2_ | 0.002 | -0.004 | 0.002 |
| ∆ Average temperature _t-3_ | 8.4e-40 | 0.011 | 8.4e-3 |
| ∆ Average relative humidity | 0.008 | 0.003 | 0.008 |
| ∆ Average relative humidity _t-1_ | 0.006 | 0.014 | 0.006 |
| ∆ Average relative humidity _t-2_ | 0.007 | 0.014 | 0.007 |
| ∆ Average relative humidity _t-3_ | 0.008 | 0.019 | 0.007 |
| ∆ Average precipitation | -0.110 | -0.140 | -0.110 |
| ∆ Average precipitation _t-1_ | -0.147 | 0.067 | -0.147 |
| ∆ Average precipitation _t-2_ | 0 | 0.659 | 0 |
| ∆ Average precipitation _t-3_ | -0.073 | 0.854 | -0.073 |
| People young older than 18 years old | -0.033 | 0.011 | -0.033 |
| Adolescent older than 12 years old | 0.022 | 0.006 | 0.022 |
| Children older than 5 years old | 0.062 | -0.015 | 0.062 |
| Use of mask | 0.0311 | 0.006 | 0.031 |
| Gathering | 0 | -0.028 | 0 |
| Stay at home | 0 | -0.014 | 0 |
| Restaurant closed | 0 | -0.007 | 0 |
| Bar closed | 0 | -4e-3 | 0 |
| ∆ Log of fully vaccinated people in any state | -0.066 | -0.212 | -0.066 |
| ∆ Log of fully vaccinated people in any state _t-1_ | -0.088 | -0.136 | -0.088 |
| ∆ Log of fully vaccinated people in any state _t-2_ | -0.021 | -0.098 | -0.021 |
| ∆ Log of fully vaccinated people in any state _t-3_ | -0.013 | -0.069 | -0.012 |
| Alpha | 0 | 0 | 0 |
| Beta | 0 | 0 | 0 |
| Gamma | 0 | 0 | 0 |
| Delta | -0.017 | 0.010 | -0.017 |
| Epsilon | -0.008 | 0.017 | -0.008 |
| Eta | -0.018 | 0.093 | -0.018 |
| Iota | 0 | 0 | 0 |
| Kappa | 0.015 | 0.011 | 0.015 |
| Zeta | 0 | 0 | 0 |
| Mu | -0.018 | -0.068 | -0.018 |
| Omicron | -0.034 | -0.059 | -0.034 |
| ∆ Log of unemployment insurance weekly claims | 0.053 | 0.129 | 0.053 |
| ∆ Log of unemployment insurance weekly claims _t-1_ | 0.007 | 0.118 | 0.007 |
| ∆ Log of unemployment insurance weekly claims _t-2_ | 0.051 | 0.149 | 0.051 |
| ∆ Log of unemployment insurance weekly claims _t-3_ | -0.038 | 0.110 | -0.038 |
| ∆ air quality index | 3e-4 | 0.001 | 3e-4 |
| ∆ air quality index _t-1_ | 3.7e-4 | 0.002 | 3.7e-4 |
| ∆ air quality index _t-2_ | -0.001 | 0.001 | -0.001 |
| ∆ air quality index _t-3_ | - 4e-4 | 3e-4 | -4e-4 |
| ∆ PM25 | 0 | 0.005 | 0 |
| ∆ PM25 _t-1_ | -0.003 | 0.006 | -0.003 |
| ∆ PM25 _t-2_ | 4.3e-5 | 0.004 | 4.3e-5 |
| ∆ PM25 _t-3_ | 0.004 | 0.001 | 0.004 |
| ∆ PM25 _t-2_ | 4.2e-4 | 0.007 | 4.1e-4 |

Abbreviation: ∆, First difference, Log, logarithm, _t-1,_ first lag, _t-2,_ second lag, _t-3,_ third lag and _t-4,_ fourth lag.

**Table S5.** Estimations of nonstationary heterogeneous panels for U.S. from January 2021 through January 2022.

|  | **Panel A**  (7-day average positivity rate) | | **Panel B**  (Population mobility index) | | **Panel C** ^d^  (Number of doses applied) | |
| --- | --- | --- | --- | --- | --- | --- |
|  | **PMG** | **MG** | **PMG** | **MG** | **PMG** | **MG** |
| **General ECM Models** | | | | | | |
| **Long-run coefficients** | | | | | | |
| Log of number of doses administered to people | -6.551*** (0.455) | 5.552 (10.052) | 0.304*** (0.086) | 11.365 (10.615) |  |  |
| Population mobility index | 1.260*** (0.142) | 13.151 (10.134) |  |  | 0.155*** (0.024) | 0.056* (0.033) |
| Average temperature |  |  | -0.012*** (0.002) | 0.202 (0.204) | - 0.013** (0.005) | 0.002 (0.005) |
| Average relative humidity | -1.266*** (0.065) | -1.213*** (0.280) | 0.077*** (0.013) | 1.185 (1.065) | - 0.165*** (0.013) | 0.114*** (0.016) |
| Constant | -16.903*** (1.196) | 11.123 (9.248) | 8.518*** (1.356) | 11.635*** (2.035) |  |  |
| Error correction coefficient | -0.270*** (0.014) | -0.317*** (0.024) | -0.187*** (0.030) | -0.305*** (0.046) | - 0.206*** (0.008) | -0.327*** (0.017) |
| No. Obs | 2681 |  | 2695 |  | 2744 |  |
| No. States | 49 |  | 49 |  | 49 |  |

Abbreviation: Log, logarithm, PMG, Pooled Mean Group estimator, MG, Mean Group estimator, Joint H-stat, Durbin-Wu-Hausman test

**Table S6.** Estimations of nonstationary heterogeneous panels (PMG) of positivity rate, mobility index and logarithm of doses vaccines applied for U.S from January 2021 through January 2022.

|  | **Panel A**  (7-day average positivity rate) | | **Panel B**  (Population mobility index) | | **Panel C** ^d^  (Number of doses applied) | |
| --- | --- | --- | --- | --- | --- | --- |
|  | **General** | **Machine learning** | **General** | **Machine learning** | **General** | **Machine learning** |
| **Long-run coefficients** |  |  |  |  |  |  |
| Log of number of doses administered to people | - 6.55*** (0.46) | - 3.59*** (0.44) | 0.30*** (0.09) | 0.29*** (0.11) |  |  |
| Population mobility index | 1.26*** (0.14) | 1.94*** (0.19) |  |  | 0.15*** (0.02) | 0.06** (0.03) |
| Average temperature |  |  | - 0.01*** (0.00) | - 0.01*** (0.00) | - 0.01** (0.00) | -0.04*** (0.00) |
| Average relative humidity | - 1.27*** (0.07) | - 1.10*** (0.07) | 0.08*** (0.01) | 0.07*** (0.02) | - 0.16*** (0.01) | -0.12*** (0.01) |
| **Short-run coefficients** |  |  |  |  |  |  |
| Error correction | - 0.27*** (0.01) | - 0.23*** (0.01) | - 0.19*** (0.03) | - 0.23*** (0.02) | - 0.20*** (0.00) | - 0.11*** (0.00) |
| ∆ Log of number of doses administered to people | - 1.68*** (0.20) | - 0.52*** (0.16) | 0.91*** (0.16) | 0.69*** (0.14) |  |  |
| ∆ Log of number of doses administered to people _t-1_ | - 1.78*** (0.22) | - 0.44*** (0.16) | - 0.15** (0.06) | - 0.16*** (0.06) | -0.03 (0.02) | 0.01*** (0.01) |
| ∆ Log of number of doses administered to people _t-2_ | - 0.83*** (0.21) |  | - 0.07 (0.07) | - 0.05 (0.06) | 0.00 (0.01) | 0.03 (0.01) |
| ∆ Log of number of doses administered to people _t-3_ | - 0.79*** (0.19) |  | - 0.06* (0.03) |  |  |  |
| ∆ Population mobility index |  |  |  |  | 0.08*** (0.01) | 0.07*** (0.00) |
| ∆ Population mobility index _t-1_ |  |  | - 0.44*** (0.04) | - 0.37*** (0.03) | - 0.01 (0.01) | -0.00*** (0.00) |
| ∆ Population mobility index _t-2_ |  |  | - 0.24*** (0.03) | - 0.16*** (0.02) | -0.05*** (0.01) |  |
| ∆ Population mobility index _t-3_ |  |  | - 0.14*** (0.03) |  | -0.05*** (0.00) |  |
| ∆ 7-day average positivity rate |  |  | - 0.06*** (0.18) | - 0.07*** (0.01) | 0.01*** (0.00) | 0.01*** (0.00) |
| ∆ 7-day average positivity rate _t-1_ | 0.88*** (0.03) | 0.79*** (0.03) | - 0.00 (0.02) | 0.01 (0.02) | 0.00 (0.00) | -0.00 (0.00) |
| ∆ 7-day average positivity rate _t-2_ | - 0.31*** (0.04) | - 0.29*** (0.04) | - 0.01 (0.02) | 0.00 (0.02) | - 0.02*** (0.00) |  |
| ∆ 7-day average positivity rate _t-3_ | 0.32*** (0.06) | 0.17*** (0.05) | 0.01 (0.01) |  | 0.01*** (0.00) |  |
| ∆ Average temperature | 0.01 (0.01) | 0.02* (0.01) | 0.04*** (0.01) | 0.03*** (0.00) | 0.00*** (0.00) |  |
| ∆ Average temperature _t-1_ | 0.05*** (0.01) | 0.05*** (0.01) | 0.05*** (0.00) | 0.04*** (0.00) | 0.00*** (0.00) | - 0.00*** (0.00) |
| ∆ Average temperature _t-2_ | 0.08*** (0.01) | 0.06*** (0.01) | 0.00* (0.00) | 0.00 (0.00) |  |  |
| ∆ Average temperature _t-3_ | 0.09*** (0.01) | 0.09*** (0.01) | - 0.02*** (0.00) |  | 0.00 * (0.00) |  |
| ∆ Average relative humidity | - 0.22*** (0.02) | - 0.18*** (0.01) | - 0.01** (0.00) | - 0.01*** (0.00) | - 0.02*** (0.00) | - 0.01*** (0.00) |
| ∆ Average relative humidity _t-1_ | - 0.09*** (0.01) | - 0.07*** (0.01) | - 0.00 (0.00) | 0.01** (0.00) | - 0.03*** (0.00) | - 0.01*** (0.00) |
| ∆ Average relative humidity _t-2_ | - 0.08*** (0.01) | - 0.08*** (0.01) | - 0.00 (0.00) | 0.01*** (0.00) | - 0.00** (0.00) |  |
| ∆ Average relative humidity _t-3_ | 0.02 (0.01) |  | - 0.01** (0.00) |  |  |  |
| ∆ Average precipitation | - 6.98*** (1.49) | - 3.57*** (0.92) |  |  | - 0.12 (0.13) | - 0.26*** (10.09) |
| ∆ Average precipitation _t-1_ | -11.68*** (1.36) | - 8.00*** (0.66) |  |  | - 0.03 (0.18) | - 0.57*** (0.13) |
| ∆ Average precipitation _t-2_ | - 2.90** (1.19) |  |  |  | - 2.21*** (0.24) | - 1.75** (0.14) |
| ∆ Average precipitation _t-3_ | - 1.93** (0.91) |  |  |  | - 0.87*** (0.06) | - 0.44** (0.50) |
|  |  |  |  |  |  |  |
| Vaccine rollout timeline for specific age groups: |  |  |  |  |  |  |
| People young older than 18 years old |  |  |  |  | - 0.34*** (0.01) | - 0.23*** (0.01) |
| Adolescent older than 12 years old | - 0.50** (0.20) | - 0.82*** (0.15) |  |  | 0.31*** (0.02) | - 0.02 (0.01) |
| Children older than 5 years old |  |  |  |  | 0.08*** (0.01) | - 0.02*** (0.01) |
|  |  |  |  |  |  |  |
| Public policy of mobility restrictions: |  |  |  |  |  |  |
| Stay at home | - 0.17 (0.31) | - 0.09 (0.18) |  |  |  |  |
| Use of mask | - 0.80 (0.77) | - 0.15 (0.12) |  |  |  |  |
| Gatherings | 0.11 (0.31) | - 0.14 (0.11) |  |  |  |  |
| Restaurant closed | - 0.22* (0.11) | - 0.04 (0.07) |  |  |  |  |
| Bar closed | - 0.01 (0.01) | - 0.01 (0.01) |  |  |  |  |
|  |  |  |  |  |  |  |
| ∆ Log of fully vaccinated people in any state _t-1_ | - 0.80 (0.70) | - 0.61*** (0.13) |  |  |  |  |
| ∆ Log of fully vaccinated people in any state _t-2_ | 0.97** (0.40) |  |  |  |  |  |
| ∆ Log of fully vaccinated people in any state _t-3_ | - 0.06 (0.30) |  |  |  |  |  |
|  |  |  |  |  |  |  |
| Timeline of the emergence of COVID-19 variants: |  |  |  |  |  |  |
| Delta | 1.10*** (0.19) | 0.60*** (0.12) |  |  |  |  |
| Epsilon | 0.27 (0.27) | 0.71*** (0.15) |  |  | 0.04** (0.02) |  |
| Eta | 0.53** (0.21) | 0.53*** (0.16) |  |  | 0.35*** (0.03) |  |
| Kappa | 0.31 (0.23) | 0.35** (0.14) |  |  | -0.51*** (0.02) |  |
| Mu | - 0.75*** (0.11) | - 0.44*** (0.09) |  |  | 0.07*** (0.01) |  |
| Omicron | 2.07*** (0.20) | 2.53*** (0.17) |  |  | -0.28*** (0.01) |  |
| Iota | 0.38** (0.16) | 0.15** (0.06) |  |  |  |  |
|  |  |  |  |  |  |  |
| ∆ Log of unemployment insurance weekly claims | - 0.70*** (0.26) | - 0.45** (0.18) |  |  |  |  |
| ∆ Log of unemployment insurance weekly claims _t-1_ | - 0.52** (0.24) |  |  |  |  |  |
| ∆ Log of unemployment insurance weekly claims _t-2_ | - 0.82*** (0.18) |  |  |  |  |  |
| ∆ Log of unemployment insurance weekly claims _t-3_ | 0.02 (0.18) |  |  |  |  |  |
|  |  |  |  |  |  |  |
| ∆ air quality index |  |  | - 0.00 (0.00) | - 0.00 (0.00) |  |  |
| ∆ air quality index _t-1_ |  |  | 0.00 (0.00) | - 0.00* (0.00) |  |  |
| ∆ air quality index _t-2_ |  |  | 0.01*** (0.00) |  |  |  |
| ∆ air quality index _t-3_ |  |  | 0.00 (0.00) |  |  |  |
|  |  |  |  |  |  |  |
| ∆ PM25 _t-4_ | 0.07*** (0.01) | 0.02** (0.01) |  |  |  |  |
| ∆ PM25 _t-5_ | 0.05*** (0.02) | 0.02 (0.01) |  |  |  |  |
|  |  |  |  |  |  |  |
| Constant | -16.90*** (1.20) | - 2.31*** (0.32) | 8.52*** (1.36) | 10.51*** (0.84) |  |  |
|  |  |  |  |  |  |  |
| No. Obs | 2681 | 2681 | 2695 | 2695 | 2744 | 2744 |
| No. States | 49 | 49 | 49 | 49 | 49 | 49 |

Abbreviations: Log, logarithm, ∆, First difference, _t-1,_ first lag, _t-2,_ second lag, _t-3,_ third lag and _t-4,_ fourth lag.

The standard errors are reported in parenthesis and probability value of the z score, with P < .05 considered significant. The superscripts ⁎⁎⁎, ⁎⁎ and ⁎ indicate significant at 1%, 5% and 10% levels, respectively.

Figure S1


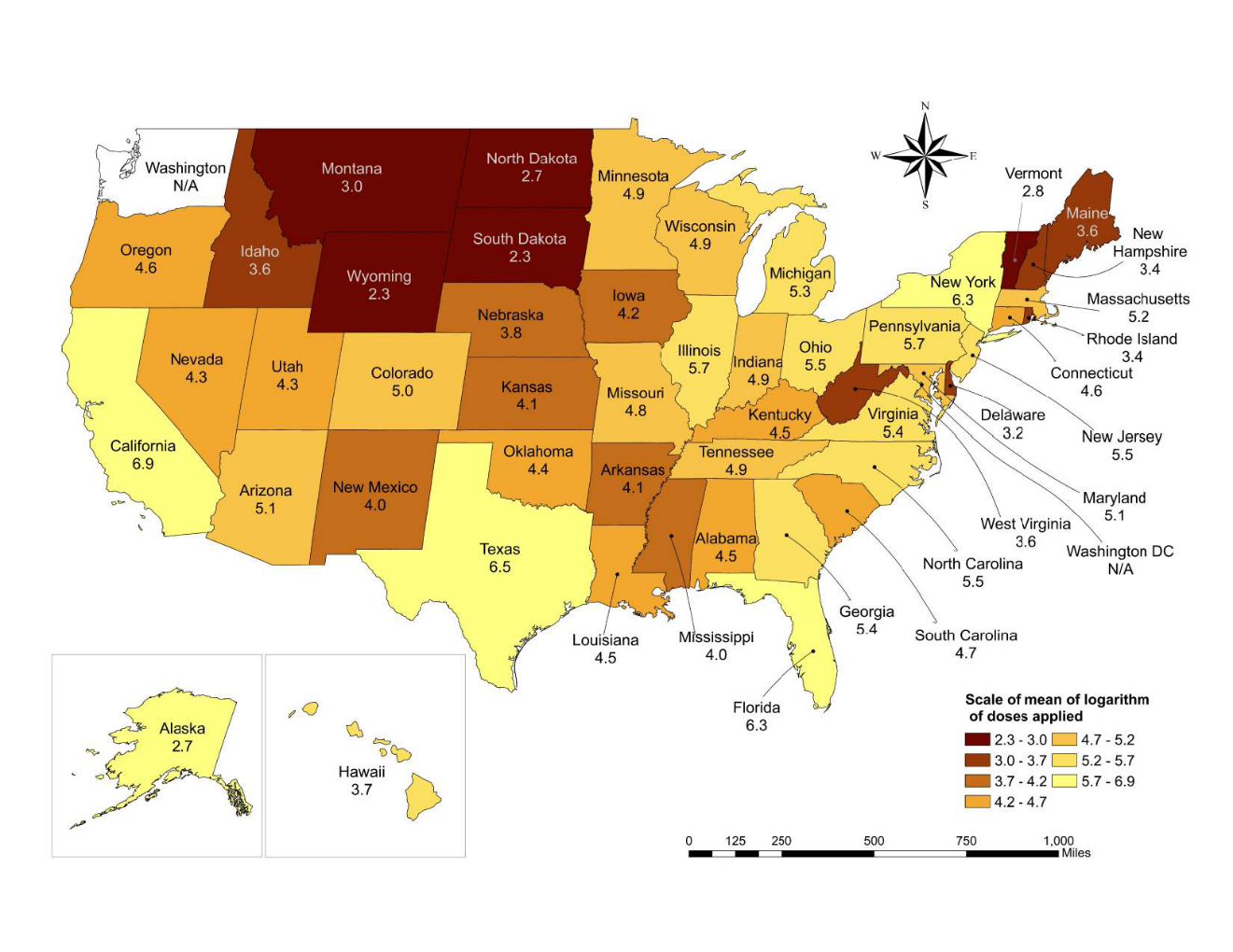


Figure S1. Logarithm of the average of doses of COVID-19 vaccines applied in the US from 1^st^ January 2021 to 23^rd^ January 2022.

Figure S2


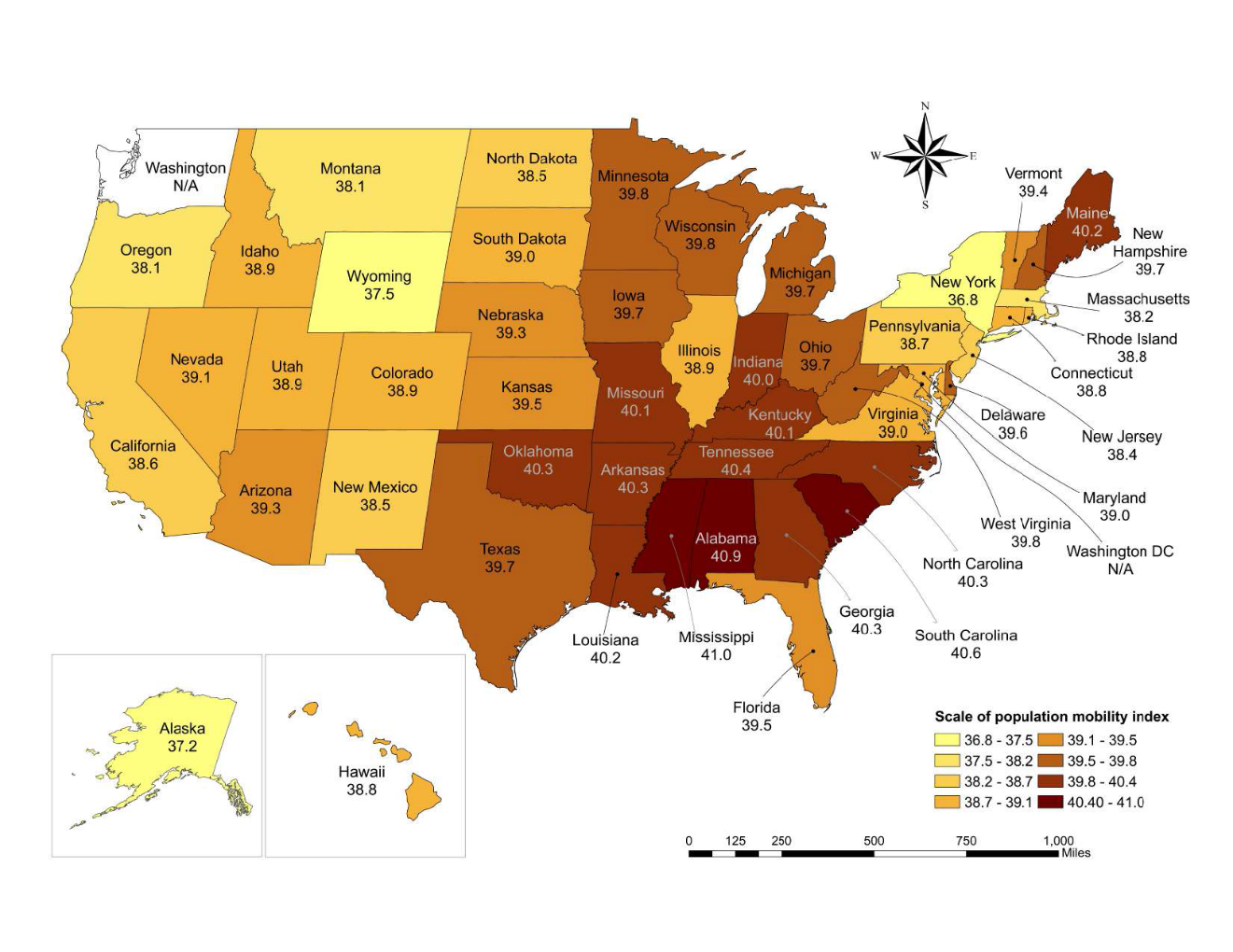


Figure S2. Average mobility index from 1^st^ January 2021 to 23^rd^ January 2022.
